# Remedies and Their Uses

**Published:** 1906-09-22

**Authors:** 


### Remedies and their Uses.

### Gujasanol.

This substitute for guaiacol, manufactured by Meister Lucius and Brüning, of Hœchst-au-Main, is a white crystalline powder, soluble in water, insoluble in alcohol and ether, having a faint odour of guaiacol and neutral reaction. It is incompatible with alkaline carbonates, but may be boiled without decomposition. The advantages claimed for this preparation are that owing to its non-toxic and non-irritating characters it can be given in considerable doses, with a correspondingly thorough therapeutic effect. Heinz showed that rabbits easily tolerated doses of forty-five grains subcutaneously, and demonstrated the occurrence of free guaiacol in the urine, showing that the drug is decomposed and guaiacol liberated within the body. Two per cent. solutions were found to have no irritant effect on the mucous membrane of the alimentary tract; 10 per cent. solutions even, though somewhat irritating, were not caustic. Its antiseptic power is much the same as that of boracic acid; it is, in adition, deodorant and slightly anæsthetic. Einhorn (M.M.W., 1900, No. 1), who has used gujasanol extensively in phthisis, emphasises the importance of adequate dosage in order to obtain a due effect. By the mouth 45 to 180 grains may be given daily at the commencement of the treatment. A concentrated watery solution may also be used for subcutaneous injection; it is easily absorbed, and even in doses of 45 to 60 grains gives rise to no local or general disturbances when administered in this manner. Not only does it exercise no irritating action on the gastric mucosa, it is said even to improve the appetite, and to be specially valuable in laryngeal and intestinal tuberculosis. Tampons of a 10 per cent. to 20 per cent. solution of the salt may be used in ozœna, the tampons remaining in position for thirty to sixty minutes. As a deodorant it may be used in ulcerating cancers, syphilitic sores, and stomatitis; and as an antiseptic in cases of pyloric stenosis with fermentation of the stomach contents. Shœfen (*Ibid.*, 1903, No. 1), while cautioning against the idea that gujasanol has any specific action, states that it is an extremely useful drug in various forms of tuberculous and septic ulceration, mainly owing to the large amounts of guaiacol which can by this means be introduced into the blood stream. One per cent. solutions may also be used in ophthalmic practice. Chemically, gujasanol is the hydrochloride of a compound formed from guaiacol diethyl and glycosol (amido-acetic acid) with the loss of one molecule of water.

$$C_6H_5.OCH_3.O[HOH]OCCH_2N(C_2H_5)_2$$

It may be conveniently prescribed in powder form, a powder to be dissolved in water before being taken.

